# Comparison of hemodynamic response to tracheal intubation and postoperative pain in patients undergoing closed reduction of nasal bone fracture under general anesthesia: a randomized controlled trial comparing fentanyl and oxycodone

**DOI:** 10.1186/s12871-016-0279-x

**Published:** 2016-11-17

**Authors:** Yeon Sil Lee, Chong Wha Baek, Dong Rim Kim, Hyun Kang, Geun Joo Choi, Yong-Hee Park, Won-joong Kim, Yong Hun Jung, Young Cheol Woo

**Affiliations:** Department of Anesthesiology and Pain Medicine, Chung-Ang University College of Medicine, 102 Heukseok-ro, Dongjak-gu, Seoul, 06973 Republic of Korea

**Keywords:** Fentanyl, Hemodynamics, Intubation, Oxycodone, Postoperative pain

## Abstract

**Background:**

The present study aimed to compare the intravenous bolus effect of oxycodone and fentanyl on hemodynamic response after endotracheal intubation and postoperative pain in patients undergoing closed reduction of nasal bone fracture.

**Methods:**

In this prospective randomized double-blinded study, 64 patients undergoing closed reduction of nasal bone fracture were randomized into one of two groups: the fentanyl group (Group F) or the oxycodone group (Group O). Each drug (fentanyl 2 mcg/kg in Group F and oxycodone 0.2 mg/kg in Group O) was administered prior to the induction of general anesthesia. Hemodynamic changes after endotracheal intubation and postoperative pain were then measured in both groups.

**Results:**

There was no significant difference in the change in mean arterial pressure and heart rate between pre-induction and post-intubation in both Groups F and O (*P* > 0.05). Postoperative pain in Group O was milder than that in Group F (*P* < 0.001); however, time to awakening from the end of operation was shorter in Group F (*P* = 0.012).

**Conclusion:**

In patients undergoing closed reduction of nasal bone fracture, oxycodone attenuates hemodynamic response to endotracheal intubation similar to fentanyl. However, oxycodone is more effective than fentanyl in improving postoperative pain.

**Trial registration:**

Clinical Research Information Service (Trial registry number: KCT0001153) on 3 July, 2014

**Electronic supplementary material:**

The online version of this article (doi:10.1186/s12871-016-0279-x) contains supplementary material, which is available to authorized users.

## Background

Endotracheal intubation during general anesthesia excites the sympathetic nervous system, leading to increased blood pressure and heart rate [1, 2]. Presently, to prevent the occurrence of these hemodynamic changes during intubation, the patient can be administered opioids, topical and intravenous (IV) lidocaine or alpha and beta blockers [3].

Fentanyl is an opioid with the short onset and action duration, and has been commonly administered prior to intubation [4]. Oxycodone is a strong mu-opioid receptor agonist and its potency is also similar to that of morphine [5]. The onset time of oxycodone is similar to that of fentanyl; oxycodone therefore can be effectively utilized to minimize a patient’s hemodynamic response to sudden stimulus such as intubation, although its action duration is long.

Closed reduction of nasal bone fracture is a short-duration surgery that needs awakening shortly after anesthetic induction. Therefore, the opioids administered prior to intubation may prolong awakening processes especially when using long acting opioids such as oxycodone. But, it’s prolonged effect is beneficial in reducing postoperative pain and promoting comfortable awakening in patients.

In this prospective randomized double-blinded study, the goal was to compare the intravenous bolus effect of oxycodone and fentanyl on hemodynamic response after endotracheal intubation and postoperative pain in patients undergoing closed reduction of nasal bone fracture.

## Methods

This study was approved by the Chung-Ang University Hospital Institutional Review Board on 2 June, 2014 and was registered with the Clinical Research Information Service (KCT0001153) on 3 July, 2014. Written informed consent was obtained from all patients.

Sixty-four patients undergoing closed reduction of nasal bone fracture were enrolled in this study. We included patients aged between 20 and 65 years and classified as American Society of Anesthesiologists (ASA) Class 1 or 2. Patients were excluded if they 1) weighed < 40 kg or > 100 kg, 2) had a body mass index (BMI) > 30 kg/m^2^, 3) were pregnant or breastfeeding, 4) had a cardiovascular or respiratory disease, 5) had renal, liver, or hematological disorders, 6) had a history of cerebral or psychiatric disease, or 7) were at high risk of aspiration or regurgitation.

All patients received an adequate explanation concerning the surgical procedure including awakening and purpose of the study. Following this, informed consent was obtained. Patients were then randomly assigned to either the fentanyl group (Group F, *n* = 32) or the oxycodone group (Group O, *n* = 32) using a random numbers table generated by PASS 11.0(NCSS, Kaysville, Utah, USA). Patient group allocations were sealed in serial numbered envelopes by an anesthesiologist who did not participate in the study. Another anesthesiologist who was unaware of the patient group allocations evaluated the patient’s airway (evaluated using the Mallampati classification) in the waiting room. Patients were unaware of their assigned group. All patients were not pre-medicated with agents such as anticholinergics, anxiolytics, or analgesics which affect postoperative pain.

All patients were transferred to the operating room. Pulse oximetry and electrocardiography was performed after placing the patients in a supine position. Non-invasive blood pressure was measured twice for all patients after a 5 min-period of stabilization. Mean values were used as a reference of preoperative blood pressure and heart rate.

The anesthesiologist who was not involved in the study and intraoperative patient care opened the group allocation envelopes. Patients in Group F were administered an IV bolus of fentanyl 2 mcg/kg 2 min before induction. Those in Group O were administered an IV bolus of oxycodone 0.2 mg/kg diluted with normal saline to prevent the anesthesiologist from distinguishing between oxycodone and fentanyl. An anesthesiologist recorded any adverse events, such as dizziness, coughing, sedation or oxygen saturation in pulse oximetry < 92 % without stimulus, which occurred before anesthetic induction.

All patients were injected with lidocaine 40 mg intravenously prior to injecting propofol 2 mg/kg. Succinylcholine 1 mg/kg was administered for muscle relaxation after loss of patient consciousness. Endotracheal intubation was performed 2 min after induction of anesthesia. The anesthesiologist evaluated the Cormack-Lehane grade and the difficulty of intubation by using subjective criteria (easy, normal, or difficult). Non-invasive blood pressure and heart rate were measured and recorded at 1-min intervals for 5 min after endotracheal intubation.

General anesthesia was maintained by 1.5 minimum alveolar concentration desflurane and 50 % air in oxygen with a constant fresh gas flow of 3 L/min. Intravenous glycopyrrolate 0.2 mg was administered when bradycardia occurred (heart rate < 40 bpm), while IV ephedrine 5 mg was administered for events of hypotension (systolic arterial pressure < 80 mmHg).

Postoperatively, desflurane administration was terminated and a total 8 L/min of fresh gas flow with 100 % oxygen was administered via an endotracheal tube. Simultaneously, ventilation was assisted such that the patients were allowed to breathe spontaneously while keeping E_t_CO_2_ between 40 and 45 mmHg. Oral suction was gently performed. The patient was not interrupted by any stimulus except for a verbal request, “Open your eyes” with the same volume every 30 s or whenever there were any even minimal movement. Extubation was performed when patients nodded their heads, opened their eyes, obeyed commands, and could breathe deeply by themselves. The anesthesia time (time from induction to extubation) and the awakening time (time from discontinuation of anesthetics to extubation) were recorded for all patients.

An anesthesiologist, who was blinded to group treatments, also, also evaluated the patient’s emergence state while awakening from general anesthesia, according to Aono’s scale: 1 = calm; 2 = not calm but easily calms down to verbal instructions, tolerable requiring ordinary fixation straps for both arms and legs; 3 = not calm despite frequent verbal instructions and moderately agitated or restless, and requires physical restraint; and 4 = combative, excited, disoriented, and strongly requires physical restraint. Classifications 3 and 4 were regarded as emergence agitation [6, 7]. Patients were subsequently transferred to post-anesthetic care unit(PACU).

Postoperative pain was evaluated for all patients in the PACU 30 min after extubation using the Numeric Rating Scale (NRS: 0 = no pain, 10 = worst pain imaginable). If the NRS score exceeded 6 or if patients requested analgesics, IV ketorolac 30 mg was administered. Patients then were sent to the ward when their modified Aldrete score were more than 9.

All the patients were also observed for 24 h postoperatively to see whether complications such as respiratory depression, sedation, nausea, or vomiting occurred. IV ketorolac was also administered if patients requested analgesics during the 24 h period postoperatively. The incidence of rescue analgesic administration was also recorded.

Data collection was performed by an anesthesiologist who was unaware of patient group allocation. The primary outcomes of this study were changes in mean arterial blood pressure (MBP) occurring after endotracheal intubation and postoperative pain in both groups. Delta MBP (ΔMBP) and delta heart rate (ΔHR) were defined as the maximum difference between non-invasive MBP and heart rate measured every 1 min during a 5-min period and the preoperative values. The secondary outcomes of this study included the incidence of emergence agitation when the patient woke up from general anesthesia, the awakening time, the incidence of rescue analgesic administration and any perioperative adverse events.

The sample size of this study was determined based on a pilot study of 20 patients undergoing closed reduction of nasal bone fracture. In this pilot study, the ΔMBP was 3.0 ± 14.5 in Group F and –10.0 ± 15.4 in Group O. In addition, the pain score was 4.1 ± 1.9 in Group F and 2.4 ± 1.8 in Group O. The number of patients required for each group was 29 in relation to ΔMBP and 26 in terms of pain, with the alpha error assumed to be 0.05 and the beta error assumed to be 0.10. We selected the maximum value of the sample size calculation to obtain an appropriate examination between the two primary end points. We assumed a dropout rate of 10 %; therefore, a total of 64 patients were included in this study.

Statistical analysis was performed using SPSS v. 21.0 (IBM Corp., Armonk, NY, USA). The normal distribution of continuous variables was evaluated by the Shapiro-Wilk test. Parametric data were analysed using the independent *t*-test, while non-parametric data were analyzed with the Mann-Whitney *U*-test. Descriptive variables were evaluated by the *χ* ^2^ test or Fisher’s exact test. Intervals prior to the administration of the first dose of rescue analgesics were assessed using the Kaplan-Meier method and the differences were evaluated by the log-rank test. All variables were expressed as median (range), mean (standard deviation), or number (%). *P*-values < 0.05 were considered statistically significant. Relative risks or median differences with 95 % confidence intervals were also calculated.

## Results

**Fig. 1:**
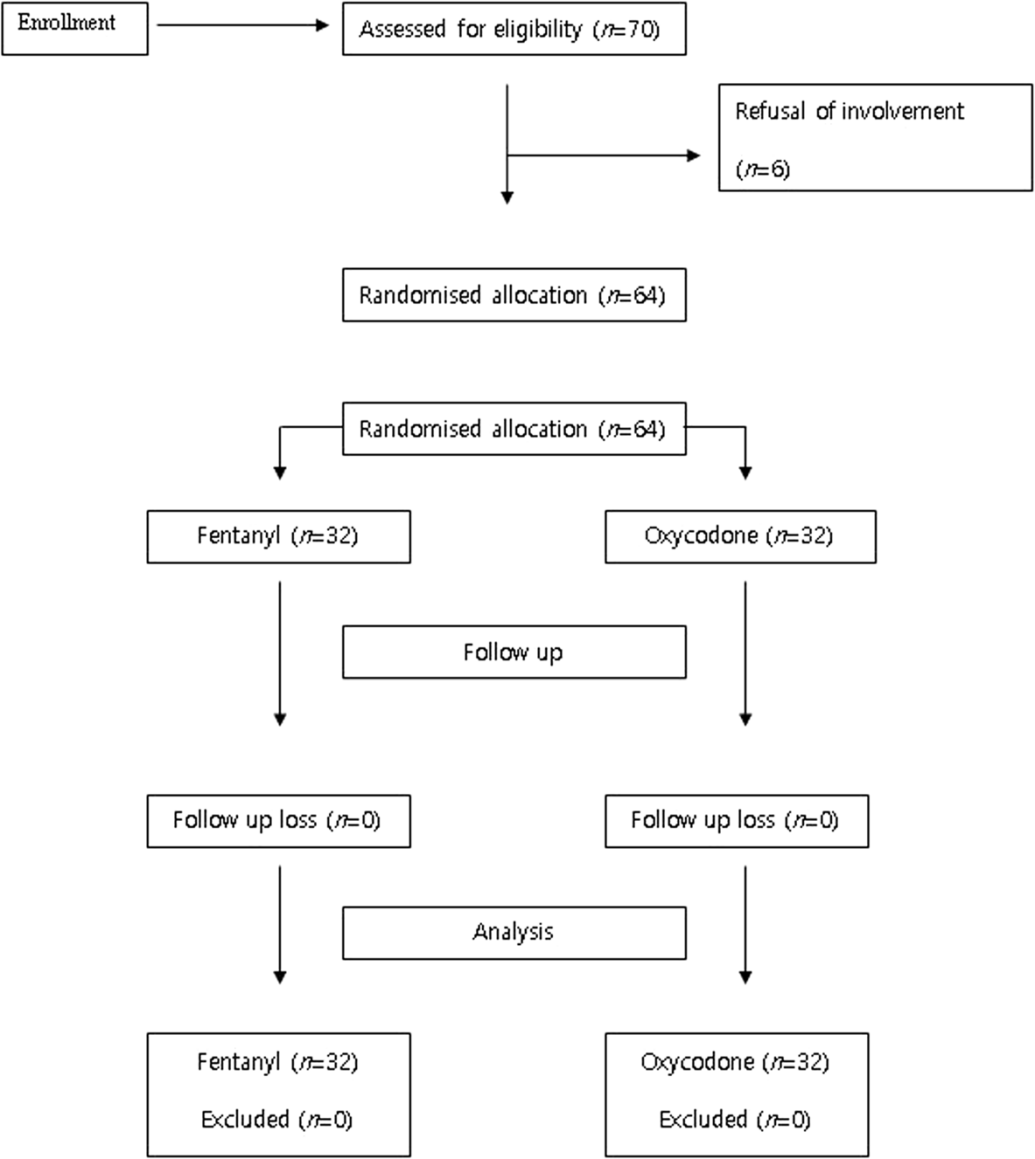
CONSORT flowchart showing the number of patients at each study phase

Data were collected between July 2014 and May 2015. A total of 64 patients were randomized into either Group F or Group O (Fig. 1). Sex, age, height, weight, BMI, ASA classification, anesthesia time, Mallampati classification, and Cormack-Lehane grade were not significantly different between the two groups (Table 1).

**Table 1:** Patient characteristics in both study groups. Values are expressed as either mean(standard deviation) or median (ranges)

	Fentanyl (*n* = 32)	Oxycodone (*n* = 32)	*P* value	RR^a^ or MD^b^ (CI^c^)
Age (yr)	28.50 (24.00–40.75)	27.50 (22.25–32.75)	0.303	1.00 (–5.41–7.41)
Sex (men/women)	23/9	20/12	0.424	0.65 (0.23–1.87)
Height (m)	1.70 (9.16)	1.68 (8.00)	0.470	1.56 (–2.74–5.86)
Weight (kg)	65.37 (12.08)	62.62 (10.04)	0.326	2.75 (–2.80–8.30)
BMI^d^ (kg/m^2^)	22.60 (2.73)	22.11 (2.57)	0.464	0.49 (–0.84–1.82)
ASA^e^ (I/II)	31/1	29/3	0.302	3.2 (0.32–32.60)
Mallampati classification (I/II/III/IV)	14/5/7/6	11/3/8/10	0.588	NA^f^
Cormack-Lehane grade (I,II,III,IV)	8/7/10/7	4/6/14/8	0.543	NA
Difficulty of intubation (easy/normal/difficult)	7/13/12	5/12/15	0.702	NA
Anesthesia time (min)	30.00 (26.25–40.00)	35.00 (30.00–40.00)	0.462	−5.00 (−11.59−1.59)

^a^ *RR* relative risk, ^b^ *MD* mean difference, ^c^ *CI* confidence interval, ^d^ *BMI* body mass index, ^e^ *ASA* American Society of Anesthesiologists, ^f^ *NA* non-applicable

ΔMBP and ΔHR were not statistically different between the groups (*P* = 0.110 and *P* = 0.950, respectively; Table 2). The number of patients whose ΔMBP increased more than 40 % compared to pre-induction levels was the same in the two groups (2/32, 6 % in each group; Table 2). The number of patients whose ΔHR increased more than 20 % was 15 in Group F (47 %; Table 2) and 17 in Group O (53 %; Table 2); the difference was not significant (*P* = 0.617; Table 2). Postoperative pain in Group O was milder than that in Group F (*P* < 0.001; Table 2).

**Table 2:** Hemodynamic changes, postoperative pain, awakening agitation, and perioperative adverse events. Values are expressed as mean(standard deviation), number (%), or median (ranges)

	Fentanyl (*n* = 32)	Oxycodone (*n* = 32)	*P* value	RR^a^ or MD^b^ (CI^c^)
ΔMBP (mmHg)^d^	12.63 (16.76)	5.51 (18.29)	0.110	7.12 (−1.64−15.89)
ΔMBP >40 %	2 (6)	2 (6)	1.000	1.00 (0.13–7.57)
ΔHR^e^	18.47 (13.74)	18.71 (16.84)	0.950	−0.24 (−7.92−7.44)
ΔHR >20 %	15 (47)	17 (53)	0.617	1.28 (0.48–3.43)
Pain	5.00 (3.00–6.75)	3.00 (2.00–4.00)	<0.001	3.00 (0.59–3.41)
Rescue analgesic	17 (53)	9 (28)	0.042	1.89 (0.99−3.59)
Awakening time (s)	335.00 (284.50–427.50)	420.00 (311.00–500.00)	0.012	−85.00 (−157.99−12.01)
Agitation (1,2/3,4)	11/21	24/8	0.001	0.18 (0.06–0.52)
Perioperative adverse events				
Cough (before induction)	5 (16)	7 (22)	0.522	1.51 (0.43–5.38)
Dizziness (before induction)	5 (16)	0 (0)	0.020	NA^f^
Oxygen saturation < 92 % (before induction)	0 (0)	4 (13)	0.039	NA
Hypotension	4 (13)	3 (9)	0.689	0.72 (0.15–3.53)
Bradycardia	0 (0)	3 (9)	0.076	NA

^a^ *RR* relative risk, ^b^ *MD* mean difference, ^c^ *CI* confidence interval, ^d^ *ΔMBP* delta mean blood pressure, ^e^ *ΔHR* delta heart rate, ^f^ *NA* non-applicable

The incidence of agitation at awakening from anesthesia was lower in Group O than in Group F (*P =* 0.001; Table 2) while awakening time was longer in Group O than in Group F. (*P* = 0.012; Table 2). There was a significant difference (*P* = 0.042) in the incidence of rescue analgesics between the two groups: 17 patients in Group F (53 %; Table 2) and nine in Group O (28 %; Table 2).

With regard to perioperative adverse events, after administering opioids and before anesthesia induction, 5 patients in group F felt dizziness compared to 0 patients in group O (*P* = 0.020). Otherwise, 4 patients in group O showed oxygen saturation in pulse oximetry < 92 % compared to 0 patients in group F (*P* = 0.039). There were no significant differences between two groups in the other perioperative adverse events (Table 2). No patients in both groups had complications including respiratory depression, sedation, nausea, or vomiting during postoperative 24 h.

## Discussion

In this study, administering oxycodone 0.2 mg/kg prior to endotracheal intubation was as effective as fentanyl 2 mcg/kg for attenuating hemodynamic response during surgery for closed reduction of nasal bone fracture. Fentanyl is a mu-opioid receptor agonist characterized by high potency, rapid onset, and short action duration [8]. Koch et al. reported that the onset time of fentanyl is about 2–3 min after IV injection [9]. It has been shown that timed delivery of low doses of fentanyl can, to some extent, counterbalance the short lasting elevation in HR and blood pressure resulting from endotracheal intubation. Sawan et al. reported that it is preferable to administer fentanyl 2 mcg/kg in patients without hypertension and fentanyl 4 mcg/kg in patients with hypertension to minimize the changes in heart rate, systolic blood pressure, and cardiac output associated with endotracheal intubation when anesthesia is induced via IV target-controlled infusion of propofol (plasma concentration, 4.0 mcg/mL) [10]. Since IV oxycodone also has a rapid onset time about 5–8 min [9, 11, 12], it could minimize patient hemodynamic responses to sudden stimuli such as endotracheal intubation, similar to fentanyl.

A typical pressor response can include a 40 % increase in blood pressure and a 20 % increase in heart rate [8]. In the present study, ΔMBP increased by more than 40 % is only two patients (6 %) and ΔHR by more than 20 % is 16 patients (50 %) in Group F. In Group O, ΔMBP increased by more than 40 % is two patients (6 %) and ΔHR by more than 20 % is 17 patients (53 %). This shows that IV administration of fentanyl 2 mcg/kg and oxycodone 0.2 mg/kg attenuated the increase in blood pressure post-intubation to a similar degree; however, their effect may be somewhat insufficient to prevent increase in heart rate. There have been some studies that indicate more opioid is required to block the elevation of heart rate than that of blood pressure [13, 14]. However, larger amounts of opioid will cause hypotension just before endotracheal intubation or after relief of excited sympathetic nervous system caused by endotracheal intubation. In this study, we therefore used 2 mcg/kg of fentanyl and 0.2 mg/kg of oxycodone which attenuates (but not totally abolish) an excessive hemodynamic response after intubation in healthy patients without cardiovascular disease.

With respect to pain, postoperative pain in Group O was milder than that in Group F. The NRS score in Group O was lower than in Group F. Fewer patients in Group O required analgesics postoperatively compared with Group F. This was probably due to the longer action duration of oxycodone than that of fentanyl. Kalso et al. also suggested that parenteral oxycodone provided faster and longer-lasting pain relief, even compared with morphine [9].

Furthermore, the incidence of emergence agitation at awakening from anesthesia was lower in Group O than in Group F. There are no clearly fixed criteria used to evaluate the occurrence of emergence agitation and its intensity in existing studies, but the Aono’s four-point scale, Riker Sedation-Agitation Scale, Richmond Agitation-Sedation Scale, or personally categorized criteria have been used [15]. We tried to reduce the bias occurred by this subjective evaluating method, and Aono’s four-point scale which was scored by a blinded anesthesiologist who had previously evaluated the agitation state of patients using this method. (or instrument or tool) [6].

In closed reduction of nasal bone fracture, there could be discomfort from the nasal packing performed to prevent postoperative bleeding [15], and this may be commonly associated with agitation. In addition, it is safer to extubate the patient when fully awake, as it could be difficult to maintain the airway due to nasal packing and the risk of aspiration from nasal bleeding. Awake extubation may be one of the causes of patient agitation during awakening. Emergence agitation is a significant concern after general anesthesia, and may lead to serious consequences for the patient including injury, increased pain, hemorrhage, self-extubation, and removal of catheters; it may also necessitate physical or chemical restraint of the patient [6]. Previous studies have suggested that postoperative pain can be a risk factor associated with emergence agitation [16].

The longer duration of action of oxycodone caused the patients in Group O to wake up later than those in Group F, by approximately 85 s in our study. However, none of the patients experienced any problems in terms of delayed awakening during 24 h postoperatively. The number of patients who showed respiratory depression after receiving drugs prior to anesthetic induction was four in Group O compared with none in Group F. In contrast, five patients in Group F experienced dizziness compared with none in Group O.

To discuss the results of this study, we have to consider the doses of fentanyl and oxycodone. The safe dose conversion ratio of IV oxycodone to IV fentanyl is yet to be established [9]. Previous studies administered a morphine to oxycodone ratio of 1:1 [17, 18] and a fentanyl to morphine ratio of 1:100 [19, 20]. On this basis, we calculated a workable fentanyl to oxycodone ratio of 1:100 for the present study. Although it was not statistically significant, ΔMBP in Group O was slightly lower than Group F. This may indicate an over dose of oxycodone. If the dose of oxycodone is reduced, it could prevent the patient from experiencing respiratory depression and delayed awakening, while still attenuating the hemodynamic response.

This study has several limitations. First, we administered oxycodone for young and healthy patients as a single dose to compare it with fentanyl. Hemodynamic response after endotracheal intubation in geriatric patients might be clinically more problematic. So, further studies on a variety of patients and dose-titrations are required. Secondly, we hypothesized that the onset time of fentanyl and oxycodone are same. We administered fentanyl or oxycodone 2 min prior to induction, so that the intubation was performed about 3 min after administration of these opioids in both groups. However, the peak effect time of oxycodone for attenuating the hemodynamic response to sudden stimulus like endotracheal intubation is, to our knowledge, not yet known, even though a study reported that the IV oxycodone showed a rapid onset of pain relief which was 5–8 min [9, 11, 12]. Thirdly, closed reduction of nasal bone fracture is not a time-consuming surgery and requires very little preparation time. In our study, there were only a few patients that required intervention to adjust the hypotension after the endotracheal intubation. There is a risk of hypotension if surgical preparation is prolonged because there is almost no stimulus while draping. Finally, succinylcholine was administered as a muscle relaxant. Succinylcholine is not frequently administered at present because it can cause hyperkalemia and rhabdomyolysis. We utilized succinylcholine because closed reduction of nasal bone fracture is a short-duration surgery. Instead of succinylcholine, rocuronium with sugammadex may be administered. However, sugammadex is an expensive drug; therefore, its cost-effectiveness should be considered [21].

## Conclusions

In patients undergoing closed reduction of nasal bone fracture, the administration of oxycodone is advantageous in terms of both attenuating the hemodynamic response to endotracheal intubation and reducing postoperative pain despite a cost of delayed awakening.

## Additional file

Additional file 1: The datasets generated and analysed during the current study. (XLSX 38 kb)

## Abbreviations

ASA: American Society of Anesthesiologists
BMI: Body mass index
MBP: Mean arterial blood pressure
NRS: Numeric rating scale
PACU: Post-anesthetic care unit(PACU)
ΔHR: Delta heart rate
ΔMBP: Delta MBP
